# Polyp segmentation with consistency training and continuous update of pseudo-label

**DOI:** 10.1038/s41598-022-17843-3

**Published:** 2022-08-26

**Authors:** Hyun-Cheol Park, Sahadev Poudel, Raman Ghimire, Sang-Woong Lee

**Affiliations:** 1 Department of IT Convergence Engineering, Gachon University, Seongnam, 13120 South Korea; 2 School of Computing, Gachon University, Seongnam, 13120 South Korea

**Keywords:** Colonoscopy, Inflammatory bowel disease

## Abstract

Polyp segmentation has accomplished massive triumph over the years in the field of supervised learning. However, obtaining a vast number of labeled datasets is commonly challenging in the medical domain. To solve this problem, we employ semi-supervised methods and suitably take advantage of unlabeled data to improve the performance of polyp image segmentation. First, we propose an encoder-decoder-based method well suited for the polyp with varying shape, size, and scales. Second, we utilize the teacher-student concept of training the model, where the teacher model is the student model’s exponential average. Third, to leverage the unlabeled dataset, we enforce a consistency technique and force the teacher model to generate a similar output on the different perturbed versions of the given input. Finally, we propose a method that upgrades the traditional pseudo-label method by learning the model with continuous update of pseudo-label. We show the efficacy of our proposed method on different polyp datasets, and hence attaining better results in semi-supervised settings. Extensive experiments demonstrate that our proposed method can propagate the unlabeled dataset’s essential information to improve performance.

## Introduction

Colorectal cancer (CRC) is one of the most prevalent causes of cancer-related deaths globally. Most colorectal cancers start as polyps which could become threatening over time and even spread to adjacent organs. Hence, detecting polyps at an early stage can help in the timely treatment of colon cancer^1^. Colonoscopy is the gold standard for detecting the location and appearance of polyp cells, which aids doctors in removing these before they develop into CRC. Thus, precise polyp segmentation is of great importance in clinical practice. It is, however, a very daunting task due to several reasons. The polyp cells have a wide variety of geometry and textures, each associated with the risk of advancing to the cancerous stage. Furthermore, the absence of adequate amount of the labeled dataset in the medical domain hinders the current research paradigm due to a high-cost annotation. A solution to this could be an approach that could leverage a vast amount of unlabeled datasets.

In recent years, artificial intelligence (AI) maneuvers in colonoscopy have achieved encouraging and promising results. Deep learning (DL) is among the widely accepted tools in the AI field and is also a subfield of machine learning (ML). In general, it is a method of extracting class-specific important features by stacking multiple nonlinear and linear blocks in deep layers, and the information is transferred between them. Especially convolutional neural networks (CNNs), a category of deep learning algorithms, have become the center of attraction in analyzing medical images, including other computer vision tasks. The foremost reason is that it takes input as a raw image and modifies them; however, it preserves spatial relationships. In medical image analysis, preserving the spatial relationship is crucial since it shows the relationship and interconnection between normal and polyp regions. Therefore, it remarkably contributed to analyzing polyp regions and eventually helped medical experts to highlight and diagnose polyp areas^2^. The utilization of such algorithms have improved polyp localization^3^, segmentation^4^, and detection^5^ task assisted by several data augmentation techniques. Usually, all DL methods require a huge dataset to learn the class-specific features. In the case of supervised learning, the dataset should have ground-truth information. Alternately, semi-supervised learning uses both labeled and unlabeled datasets.

Recently, deep learning-based supervised methods such as FCN^6,7^, U-Net^8^ and its variants: U-Net++^9^, ResUNet++^10^, A-DenseUNet^11^ have often achieved superior performance in polyp segmentation. The success of prior methods depends on a large number of labeled data and transfer learning. With this, they can obtain the precise segmentation result; however, the performance can still suffer when dealing with polyp varying in shape, scale, and size. The presence of a massive number of the labeled dataset could solve this problem. Recently, Hyper-Kvasir-SEG^12^, the largest image and video dataset containing a gastro-intestinal track, was released to provide a positive direction towards solving the data scarcity in the medical domain. However, it comes with an exorbitant amount of unlabeled dataset, and obtaining high-quality labeling data is very expensive in clinical settings. The semi-supervised learning (SSL) aims to solve the above problems by learning from the less labeled data and unlabeled data, which is highly demanding and can impact the medical imaging research community. The semi-supervised methods have been widely studied and accepted over the years. Lee, D.-H. et al.^13^ introduced pseudo-labeling approach for the deep learning methods. First, it trains the model with labeled training set, predicts the results on the unlabeled set and then use the same predicted results with the combination of original training sets to retrain the model. Berthelot, D. et al.^14^ proposed Mix-Match to generate more accurate pseudo labels by taking average predictions of augmented inputs. The same author proposed Remixmatch^15^ by using more augmentation strategies and tackling the distribution alignment issue. Besides psdueo-labeling, current methods in SSL includes consistency training^16–18^ , entropy minimization^19^ and bootstrapping^20^. $\pi$-model was proposed which encouraged consistent prediction over two perturbed version of same input image^17^. Such technique thus works as a supervision for unlabeled set and can be easily integrated into training loss. SSL models based on generative adversarial networks have also received much attention these days^21,22^. However, the research field involving SSL has been limited to classification tasks. Its application in image segmentation is also severely limited; especially polyp segmentation has not been explored much.

This paper proposes a semi-supervised method for polyp image segmentation based on the cross-consistency regularization method and continuous update of pseudo-label generated by the teacher-student model. Our main motive is to answer the complication of insufficient training data and exorbitant labeling cost in the medical world. We propose a powerful encoder-decoder architecture for the segmentation task that achieved benchmark performance in the Medico2020 Challenge, winning first prize. We apply the mean teacher-student model concept leveraging the consistency regularization method. We randomly perturbed the unlabeled data and fed it to the teacher model, which is the student model’s exponential moving average weight. With the cross-consistency, the aftermath of cross-entropy loss of labeled data from the student model and the teacher model’s unsupervised loss is added to obtain a better model. To utilize the pseudo label, we propose to combine the continuous update of pseudo-label (CUPL) generated by the teacher-student model so that only the confident parts are used. This method can generate better pseudo-labels with the iterative optimization method and eventually achieve significant performance gain in polyp image segmentation.

The main contribution of this paper are:

- We propose encoder-decoder method that is well suited for the polyp which have varying shape, size and scales.
- We present a new and robust semi-supervised method for medical image segmentation especially for polyp images that utilizes small number of labeled images and large number of unlabeled images.
- We propose an enhanced consistency regularization method to utilize unlabeled data and encourage the model to perform consistent predictions for the same input under different perturbations.
- We propose continuous update of pseudo-label generated by the average of teacher-student model to obtain confident pseudo-labels and finally improve the performance of polyp images.
- Extensive experiments demonstrate that the proposed method achieves a good performance and lead existing method by a large margin, on two challenging datasets.

## Related work

### CNN-based polyp segmentation

Accurate polyp segmentation is crucial for the patient to reduce the overall death ratio caused by the cancer. U-Net^8^ have been widely accepted for myriads of medical segmentation tasks, which originally based on encoder-decoder architecture. Recently, various U-Net variants have been proposed to improve the segmentation performance^4,9,23–29^. HarDNet68^29^.For the automatic polyp segmentation task, several representative networks were also developed to improve the polyp segmentation perfor-mance from different aspects, including U-Net++^9^, PraNet^28^ and HarDNet-MSEG^29^. ResU-Net applies residual blocks to supplement the location information of polyps, while HarDNet-MSEG consists of the encoder of HarDNet68^30^ and the decoder of Cascaded partial decoder with receptive field block to improve both accuracy and inference speed. Besides, PraNet adopted three reverse attention modules with a parallel partial decoder connection to strengthen the area-boundary constraint for polyp segmentation. However, these methods are based on fully-supervised training strategies. Fully-supervised methods usually require sufficient labeled medical samples for training, but annotating medical data such as polyp images is often expensive and time consuming. In this regard, semi-supervised segmentation method is a better direction to achieve satisfying accuracy for polyp segmentation from limited labeled images.

### Semi-supervised training

Due to the lack of labeled images for training, semi-supervised methods turn to leverage unlabeled data to obtain useful information. Prior semi-supervised methods mainly focus on hand-crafted features to segment medical images^31–33^. A semi-supervised method was proposed for automated classification of skin cancer^31^. The authors employed deep belief neural net and support vector machine (SVM) to train the model accompained by labeled and unlabeled datasets. For the skin lesion segmentation task, Jaisakthi et al.^33^ proposed two stage methodology which includes preprocessing and segmentation stage. They determined the color of the skin lesion using histrogram and later K-means clustering is performed to segment the group of pixels of same color. Gu et al.^32^ proposed semi-supervised learning for biomedical image based on forest oriented super pixels (voxels). However these methods relies on hand-crafted features, hence lacks strong representation capability.

Recent works includes deep learning based approach for semi-supervised segmentation task. Bai et al.^34^ proposed a fully convolutional network for cardiac segmentation of MR images where network parameters and segmentation of unlabeled data is updated alternately. Similarly, pseudo labeling method^13^ also successfully extracted useful information from the unlabeled data to enhance the model training. Li et al.^21^ proposed a semi-supervised network for the skin lesion segmentation task, which only used 15 $\%$ labeled images and obtained a similar performance of several fully-supervised methods. several representative adversarial learning methods^21,22,35^ were also proposed to improve the performance of segmentation networks. Using the GAN, an extensive realistic fake images can be created by the generator and it helps discriminator to learn better feature representations accurately which eventually helps in pixel classification. Hung’s method^35^ employed the output of a fully convolutional discriminator as supervisory signals, which is combined with self-taught learning framework to provide more useful pseudo labeling information for semi-supervised training. Zhang, Y. et al.^36^ proposed adversarial-based network that utilized unannotated dataset while training networks and generated better generalization results. Attention-based GAN approach was proposed to select the confident regions of the unlabeled dataset to train the segmentation model^37^. A novel semi-supervised method was proposed for retina vessel segmentation where a GAN is used to integrate information leaking and traditional mean-teacher frameworks^38^. Another state-of-the-art technique includes Mean-Teacher, a method where teacher model’s output is calculated by using exponential weighted average of the student model^18,39–43^. In this work, we explore the mean-teacher paradigm to improve the segmentation performance leveraging unlabeled data.

**Figure 1:**
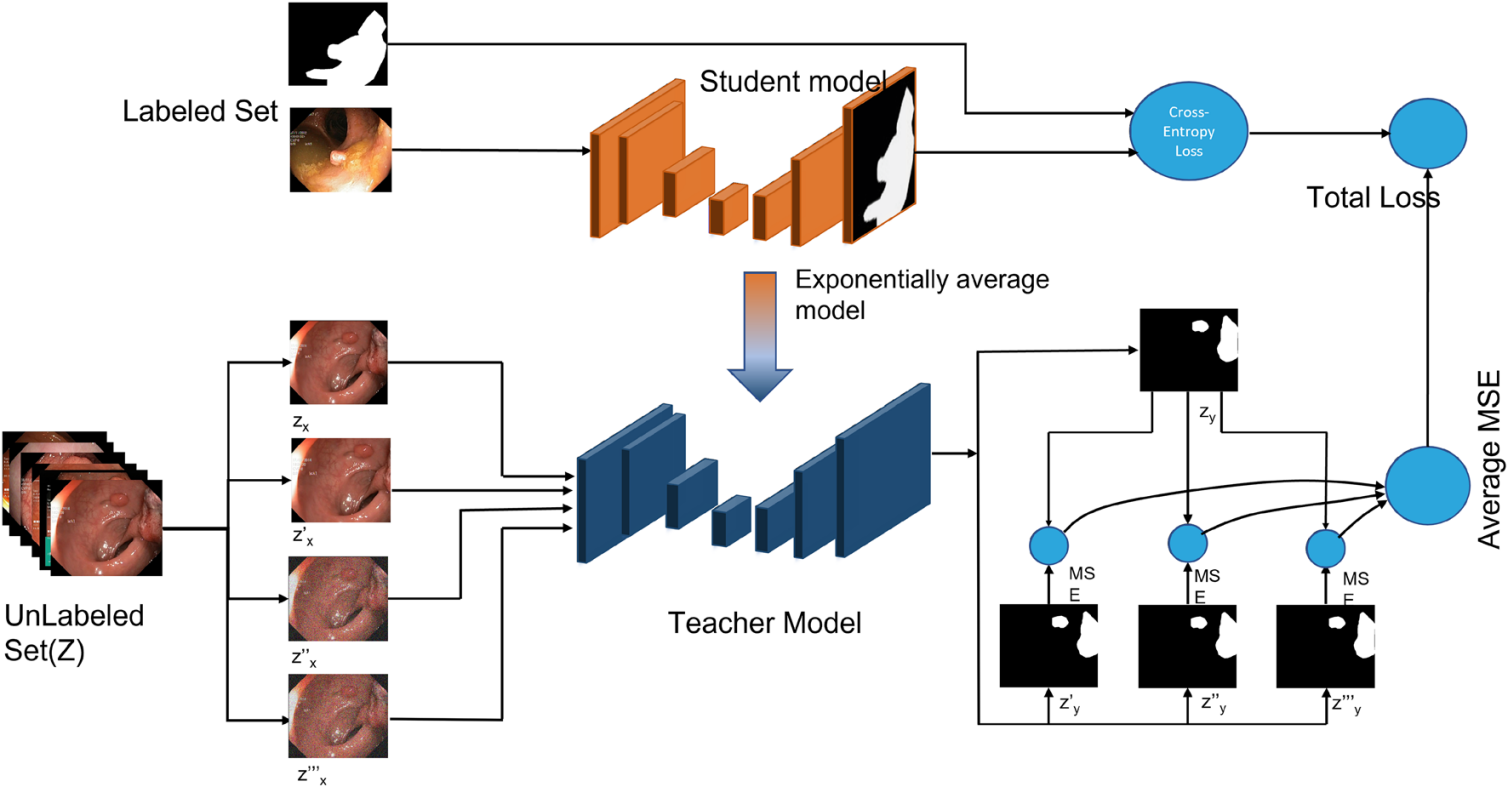
Our proposed method for semi-supervised medical image segmentation (we utilize Kvasir-SEG data^44^ as an example). The weight of the teacher model is the exponential moving average (EMA) of the student weights. The total loss is a weighted combined loss of the cross-entropy on labeled data and mean-square error (MSE) on the unlabeled set. Note that we apply transformation-consistent approach on the unlabeled data during perturbations^45^.

## Methodology

Figure 1 shows our proposed method, which employs encoder-decoder-based efficient UNet based on teacher-student model with consistency regularization method. Each modules are explained in “Related work”.

### Semi-supervised framework formulation

In semi-supervised learning, the training set consists of N inputs with X labeled sets and N-X (Z) unlabeled sets. We indicate the labeled set as X = {($$x_{1},y_{1}$$),($$x_{2},y_{2}$$).........($$x_{n},y_{n}$$)} with its corresponding mask and the unlabeled set as Z = $${z_{1} \ldots z_{n}}$$. The input 2D image is indicated by: $$x_{i} \in R^{H*W*3} $$ and ground-truth segmentation mask is $$y_{i} \in {0,1}^{H*W}$$.

The key motivation behind utilizing the semi-supervised approach for the polyp segmentation is based on the smoothness assumption, i.e., data points identical to each other in the image space are more likely to share a similar label^17,46^. These methods focus on regularization loss and different perturbations, which encourages the model to generate consistent output under different input data perturbations. By leveraging this idea, we design our networks by keeping different perturbations (random scaling, Gaussian noise, rotation) to give smooth outputs. As aforementioned, we employ a teacher-student learning mechanism for the semi-supervised task. We use cross-entropy loss function to train the student model so that it evaluates and corrects the network output on the labeled dataset X. We evaluate the teacher model on two predictions under different perturbations and take the average of all mean-squared errors. As the teacher and student model share the same network, we only train the student model and update the teacher model’s weight using the exponential moving average (EMA) of the student model. Let us define the weight of the teacher and student model as $\theta '$ and $\theta$; then the weight is updated by:

1 $$\begin{aligned} \theta ' = \alpha \theta '_{t-1}+(1-\alpha )\theta _{t} \end{aligned}$$

where $\alpha$ is the smoothing coefficient hyperparameter, which defines how the teacher model relies on the student model. A high value of $\alpha$ indicates that the teacher model is relying on its last teacher model in last step. Otherwise, the model relies on the parameters of current student model. According to the experimental settings in^18^, keeping the value $\alpha$ = 0 is equivalent as a variation of $\pi$-model and performance is better when kept $\alpha$ = 0.999. Therefore, we also follow this experimental evidence and set aforementioned values for all experiments. For the supervised segmentation applied in the student model, we use a binary cross-entropy loss to train as follows:

2 $$\begin{aligned} L = -\sum _{i}^{j}y_{i}(logy_{i}'-(1-y_{i})log(1-y_{i}')) \end{aligned}$$

L is the loss for prediction $y'$ consisting of j pixels at a specific network output. Similarly, for the consistency regularization, the teacher model predicts the two different label under different perturbations from the unlabeled dataset, and finally calculates the average of the mean-square error difference of each output. Let us suppose ${z_{y}}$ be an output of the teacher model from the unlabeled set and ${z_{y}'}$, ${z_{y}''}$, ${z_{y}'''}$ are outputs after applying different perturbations such as random scaling, Gaussian noise, and rotation of input image, and the consistency loss is applied by:

3 $$\begin{aligned} \begin{aligned} Consistency \ Loss (CL) = Average (||z_{y} - z_{y}'||^2 + ||z_{y} - z_{y}''||^2 + ||z_{y} - z_{y}'''||^2) \end{aligned} \end{aligned}$$

We apply the transformation-consistent method to utilize the unlabeled data in the unsupervised regularization^45^. The overall loss function is defined as:

4 $$\begin{aligned} Overall \ Loss = L + \lambda (CL) \end{aligned}$$

Finally, we train the model by reducing the weighted combination of supervised cross-entropy loss and the unsupervised regularization loss. Significantly, the model’s generalization capacity will be increased, and make consistent prediction by minimizing equation 3 accordance with the smoothness assumption.

### Encoder–decoder network overview

**Figure 2:**
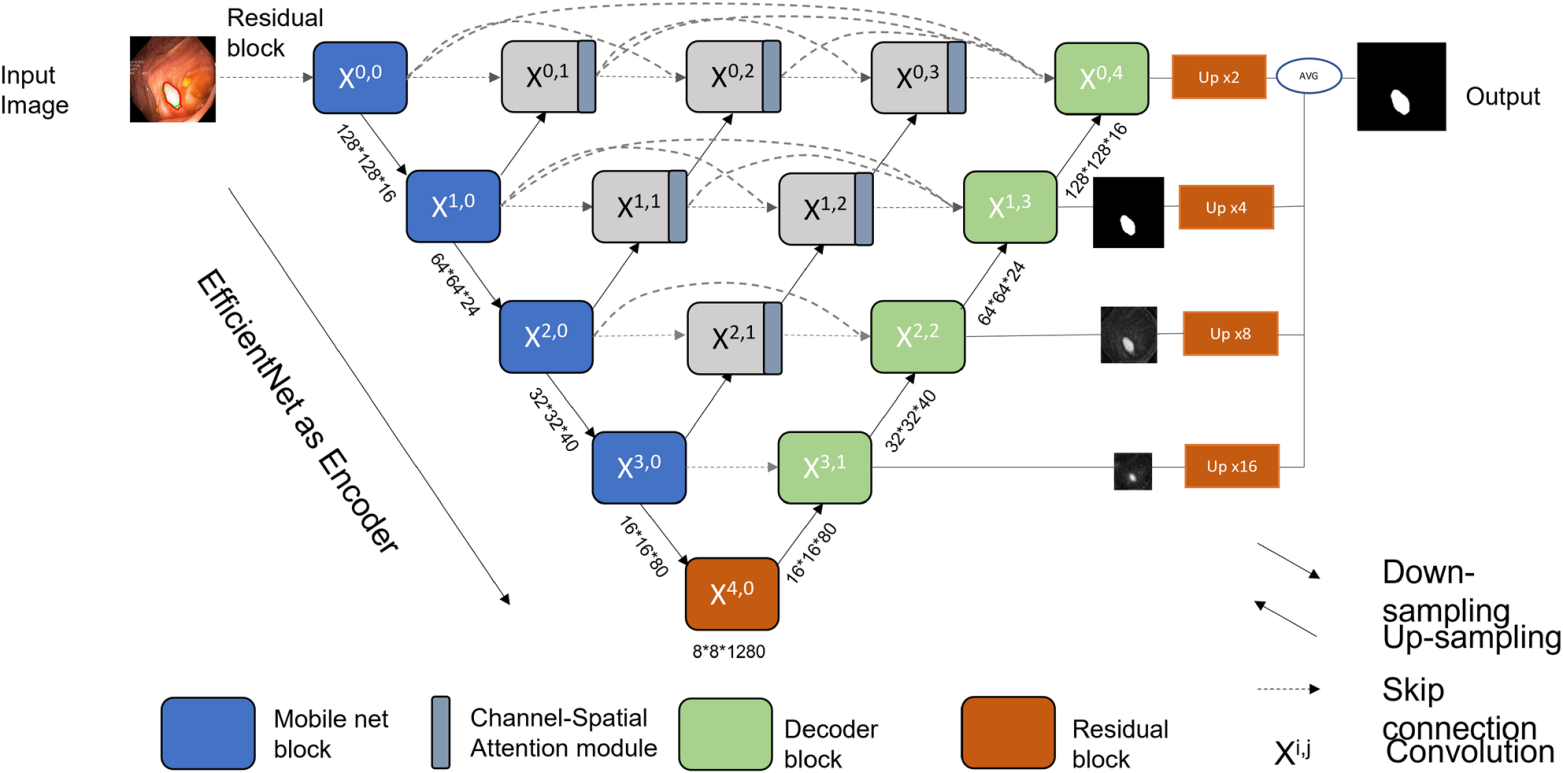
Overview of the encoder-decoder-based EfficientUnet method.

The architecture of our encoder-decoder-based UNet is shown in Fig. 2. We propose a powerful framework to enhance the strong feature representations for polyp segmentation. For the encoder path, we employ the pre-trained weight of EfficientNet^47^. The combined components such as MobileNet inverted block (MB)^48^ and squeeze and excitation network^49^ make EfficientNet as a better feature extractor. To deal with the presence of polyps of varying scales, we leverage the redesigned skip connections from the UNet++ that enables multi-scale feature fusion at the same resolution^9^.

At different levels, each node concatenates the feature maps from its previous node of the same level and the upsampled feature maps of the next level, enabling aggregation of multi-scale features. Next, the concatenated features are passed through the channel-spatial network^50^ at each node which restrains irrelevant features and allows only useful spatial details. The addition of deep supervision enables significantly better performance and faster convergence.

On the decoder side, a transposed convolution is used for upsampling the feature maps. Similarly, we upscale the outputs of the decoder block at different level and apply a 1x1 convolution with 1 kernel and a sigmoid function. Then, all the outputs (after deep supervision) are averaged and a final result is generated. With this, the model can aggregate the multi-scale semantic features and eventually increase the segmentation accuracy.

### Continuous update of pseudo-label

Pseudo-labeling is a crucial step in semi-supervised learning. This step is an improvement over our baseline model which eventually helps in generating better output masks. Initially, we train the semi-supervised method until it converges on the provided labeled X and unlabeled set Z. Then, we generate the pseudo-labels from the teacher model. We then take an average between current generated pseudo-labels with the last epoch pseudo-labels and finally add them with the original labeled datasets. We used this technique continuously to improve the segmentation accuracy on unlabeled dataset. We employ the labeled dataset X (*x*, *y*) and the unlabeled set Z as training set to the network. For the training, we denote unlabeled input 2D image by: $$z_{i} \in R^{H*W*3} $$ and ground-truth segmentation mask by $$u_{i} \in {0,1}^{H*W}$$. While generating pseudo-labels, only those images were taken and performed averaging whose outputs have the low MSE error difference between the teacher and student model so that only the confident part can be used for the ground-truth generation. The main difference with the traditional pseudo-label technique was that we keep updating the pseudo-labels by taking averages of current and last pseudo-labels in regular interval.

The loss functions after combining the labeled data and pseudo-labels of unlabeled data is as follows :

5 $$\begin{aligned} \begin{aligned} Combined \ Loss =\frac{1}{|X|}(L) + \frac{1}{|Z|}\left(-\sum _{i}^{j}u_{i}(logu_{i}'-(1-u_{i})log(1-u_{i}'))\right) \end{aligned} \end{aligned}$$

We obtain more accurate and smooth pseudo labels after continuous iterations. The whole process of semi-supervised polyp image segmentation method based on CL and CUPL is shown in Algorithm 1.

## Experiments

### Datasets and baselines

We perform experiments on two different polyp datasets: Kvasir-SEG^44^, and CVC-ClinicDB/CVC-612^51^. The Kvasir-SEG and CVC-ClinicDB/CVC-612 dataset includes a total of 1000 polyp and 612 polyp images with their corresponding ground truth respectively. We compare our proposed method with different medical image segmentation methods in semi-supervised settings. In the experiments, we utilize different portions of the dataset for comparison. For the Kvasir-SEG dataset, we utilize 50, 200, and 400 sets of labeled data. Similarly, 1/8,1/4, and 1/2 portions of labeled data were used for training in the case of the CVC-612 dataset. Further, we also perform experiments on different volume of label and unlabel data to evaluate the performance when introducing more unlabel data. All the experiments reported on the table are averaged for three trials.
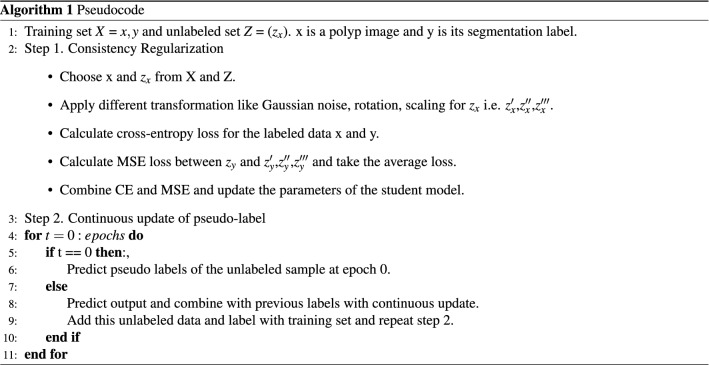


### Implementation details

We split the dataset into training, testing, and validation set with a ratio of 80:10:10 percent, respectively for both datasets. All the images are resized to 256 × 256 to reduce the computational cost and balance the segmentation performance. We implement our model in Pytorch and conduct our experiments on NVIDIA TITAN RTX GPU. As mentioned above, we employ pre-trained network EfficientNet as an encoder backbone; therefore we use Adam optimizer with a small learning rate of 0.00001 for all the experiments. Setting the high learning rate may cause undesirable divergent behavior in the loss function especially when using pretrained networks. We train both supervised and semi-supervised approach for 200 epochs. Meanwhile, we use a batch size of 40 for both supervised and semi-supervised settings. To propagate the unlabeled information using the CUPL approach, we update the pseudo-labels at every 10 epochs.

### Experiments on Kvasir-SEG dataset

#### Quantitative results with different labeled/unlabeled data

We present the quantitative and qualitative performance of the proposed method which is trained in different semi-supervised data distribution. The different labeled and unlabeled sets are randomly selected from the dataset. Table 1 presents the experimental results of different labeled and unlabeled distribution sets of training data with the baseline supervised method, Consistency loss (CL), and Continuous update of a pseudo label (CUPL) on the testing subset.

**Figure 3:**
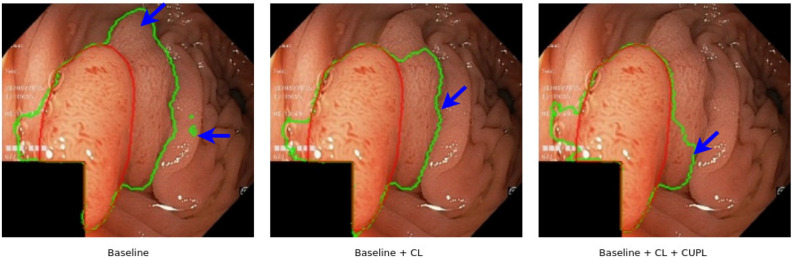
Some pseudo-labels of polyp segmentation obtained by CL and the CUPL on the validation subset.

**Figure 4:**
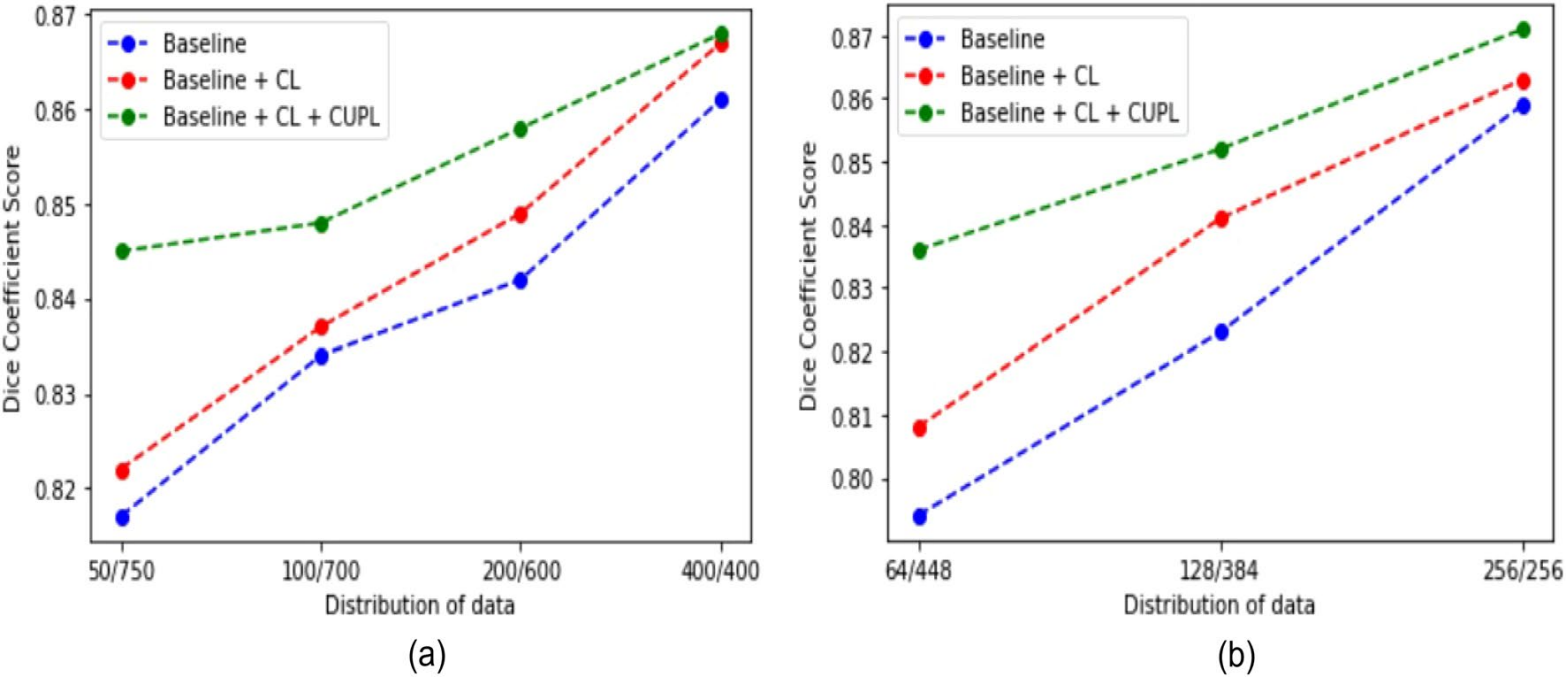
The Dice coefficient score under different distribution of labeled/unlabeled sets in (**a**) Kvasir-SEG and (**b**) CVC-612 dataset.

We apply the same network backbone while performing experiments. We use the cross-entropy loss function for the supervised training on the 50/100/200/400 sets. Further, the proposed method (combination of baseline, CL, and CUPL) is trained semi-supervised with the combined loss function as stated in Eq. (5). From Table 1, we can observe that our proposed method achieves higher segmentation accuracy in terms of all evaluation metrics with a good marginal lead over the baseline supervised model. It can be seen that baseline supervised model with the addition of CL and CUPL method increases the overall segmentation accuracy. The continuous improvements of “Baseline + CL ” and “Baseline + CL + CUPL” in Table 1 indicate that consistency loss and updating the pseudo-labels in a certain interval of time is also an effective way to increase accuracy. Figure 3 presents the pseudo-labels generated by each modules including the proposed method (Baseline + CL + CUPL). Figure 4a shows the qualitative results of different methods. Compared to the baseline supervised method, “Baseline + CL” and the proposed method generate an output that fits closely with the ground truth. Similarly, Fig. 4b shows the Dice coefficient score of the “Baseline”, “Baseline + CL” and the proposed method trained with different sets of labeled and unlabeled images. We can observe that the proposed method consistently improves the performance in different settings and demonstrates that the proposed method utilizes the unlabeled data effectively. As anticipated, the baseline supervised model’s performance is increased with an increasing number of labeled datasets. Also, the accuracy of the semi-supervised methods is increased with more labeled images (see Table 1). However, the margin gap between the baseline supervised and the proposed method decreases when the number of labeled datasets increases, indicating that the proposed method behaves well and achieves high performance when the number of labeled data is small. The improvement in accuracy indicates that consistency loss applied also acts as a regularization to the labeled dataset and encourages the model to learn the features more efficiently.

**Table 1:** Segmentation accuracy (Jaccard, Dice coefficient, Accuracy, Recall and Precision) comparison of CL and CUPL in our method on Kvasir-SEG dataset.

Label/Unlabel	Methods	Dice	Jaccard	Accuracy	Recall	Precision
50/750	Baseline	0.817	0.717	0.946	0.851	0.979
	Baseline + CL	0.822	0.725	0.943	0.852	0.975
	Baseline + CL + CUPL (proposed)	0.845	0.749	0.951	0.891	0.961
100/700	Baseline	0.818	0.716	0.945	0.900	0.959
	Baseline + CL	0.820	0.728	0.947	0.886	0.966
	Baseline + CL + CUPL (proposed)	0.846	0.744	0.942	0.921	0.944
200/600	Baseline	0.842	0.750	0.955	0.910	0.964
	Baseline + CL	0.849	0.762	0.957	0.892	0.972
	Baseline + CL + CUPL (proposed)	0.858	0.787	0.959	0.923	0.964
400/400	Baseline	0.861	0.772	0.961	0.920	0.970
	Baseline + CL	0.867	0.786	0.962	0.915	0.974
	Baseline + CL + CUPL (proposed)	0.868	0.793	0.967	0.926	0.958

#### Effectiveness of different augmentation strategies

To show the effectiveness of the different augmentation strategies in consistency regularization, we performed ablation studies on the Kvasir-SEG as shown in Table 2. The experiments were performed on 50 labeled and 750 unlabeled images and inference on the testing dataset. In the supervised settings, we trained the model on only 50 images. In Table 2, “Baseline” indicates the normal supervised learning, whereas “Baseline + CL” indicates the adoption of consistency regularization loss in training. As shown in the table, different data augmentation techniques such as random scaling, Gaussian noise and rotation contribute to the increase in performance. However, combining all three techniques enhanced the performance compared to independent ones.

**Table 2:** Ablation of semi-supervised method (50 labeled / 750 unlabeled) on the testing set of Kvasir-SEG dataset.

Methods	Dice	Jaccard	Accuracy	Recall	Precision
Baseline	0.817	0.717	0.946	0.851	0.979
Baseline + CL (RS)	0.819	0.718	0.932	0.861	0.958
Baseline + CL (GN)	0.816	0.712	0.931	0.850	0.973
Baseline + CL (R)	0.821	0.719	0.949	0.856	0.976
Baseline + CL (RS + GN)	0.821	0.720	0.952	0.859	0.967
Baseline + CL (RS + R)	0.822	0.722	0.949	0.854	0.961
Baseline + CL (GN + R)	0.822	0.721	0.953	0.862	0.942
Baseline + CL (RS + GN + R)	0.822	0.725	0.943	0.852	0.975

*RS* random scaling,* GN* Gaussian noise,* R* rotation of an image.

**Figure 5:**
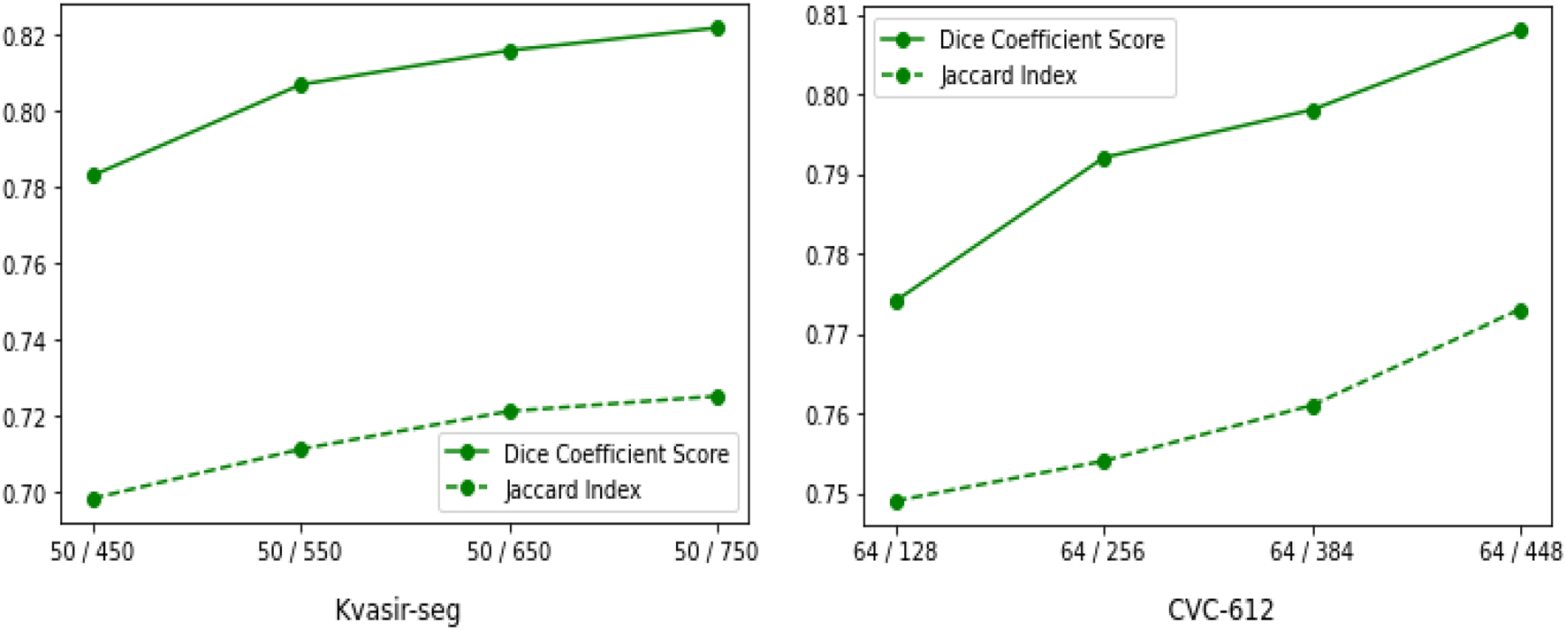
Results on the testing subset of both Kvasir-SEG and CVC-612 dataset with fixed number of labeled and different number of unlabeled images.

#### Results under different number of unlabeled data

We also perform an experiment to evaluate the model’s performance when introducing more unlabeled data. We draw the Dice coefficient score and Jaccard index in Fig. 5 of both datasets. Note that the experiments were performed on the fixed number of labeled images 50 and 64 for Kvasir-SEG and CVC-612, respectively. Similarly, a varying number of unlabeled images (450, 550, 650, 750 for Kvasir-SEG) and (128, 256, 384, 448 for CVC-612) were used for the experiments. We can observe the significant performance gain when increasing the number of unlabeled images, demonstrating that the proposed method utilizes the unlabeled data information effectively.

### Experiments of CVC-612 dataset

We show the performance of the proposed method on CVC-612 datasets to demonstrate the effectiveness of our semi-supervised method in Table 3. We split the training images as aforementioned and used the small portion of the dataset for the training purpose in semi-supervised settings. Usually, we set 64/128, 128/385/ 256/256 distributions and perform training under the same settings as the Kvasir-SEG dataset. We employ the Dice coefficient score, Jaccard index, accuracy, recall, and precision for the evaluation. We present the quantitative results in Table under different labeled/unlabeled sets. We can observe that the proposed method achieves good performance in all settings with a 2–3% improvement in dice coefficient score.

**Table 3:** Segmentation accuracy (Jaccard, Dice coefficient, Accuracy, Recall and Precision) comparison of CR and CUPL in our method on CVC-612 dataset.

Label/Unlabel	Methods	Dice	Jaccard	Accuracy	Recall	Precision
64/448	Baseline	0.794	0.751	0.918	0.874	0.956
	Baseline + CL	0.808	0.773	0.935	0.897	0.942
	Baseline + CL + CUPL (proposed)	0.836	0.789	0.939	0.916	0.948
128/384	Baseline	0.823	0.764	0.921	0.925	0.962
	Baseline + CL	0.841	0.779	0.934	0.924	0.967
	Baseline + CL + CUPL (proposed)	0.852	0.796	0.933	0.923	0.958
256/256	Baseline	0.859	0.785	0.943	0.912	0.973
	Baseline + CL	0.863	0.803	0.958	0.898	0.957
	Baseline + CL + CUPL (proposed)	0.871	0.814	0.960	0.918	0.968

### Comparison with other semi-supervised segmentation approaches

We compare the proposed method with different semi-supervised segmentation methods adopted in medical domain^34–36,45^ and present in Table 4. Note that the Mean-Teacher method^18^ is similar to our method “Baseline supervised + CL”; however, consistency loss is not included, and only the exponential average weight of the model is used for the prediction. We implement all methods mentioned above with their original settings and evaluate them on Kvasir-SEG and CVC-612 datasets. For the Kvasir-SEG dataset, we utilize 50 labeled and 750 unlabeled images. Similarly, we use 64 labeled images and 448 unlabeled images for CVC-612 images. Table 3 shows the dice coefficient score on different methods on the testing set. Compared to prior methods, the proposed method achieves the highest dice coefficient score under the settings mentioned above. The evaluation shows the effectiveness of the proposed method in comparison to prior semi-supervised methods.

**Table 4:** Comparison of different semi-supervised methods performance in Dice coefficient score.

Methods	Kvasir-SEG		CVC-612	
	Dice	Improvement	Dice	Improvement
Baseline supervised	0.817	–	0.794	–
Mean Teacher^18^	0.819	0.002	0.797	0.003
DAN^36^	0.826	0.009	0.805	0.011
GAN^35^	0.834	0.017	0.816	0.022
Self-training^34^	0.837	0.002	0.819	0.025
TCSMV2^45^	0.841	0.024	0.828	0.034
Proposed Method	0.845	0.028	0.836	0.042

## Discussion

In the medical image domain, supervised learning has been proven effective for many tasks such as classification, detection, and segmentation. However, obtaining a good performance depends on the amount of dataset availability. Therefore, suggesting new methods that require limited ground-truth data will benefit the clinical world. In this manuscript, we propose a semi-supervised-based deep learning framework that takes advantage of the unlabeled dataset and efficiently reduces the annotation effort of large-scale datasets. The primary insight of our proposed method is the adoption of consistency training with continuous updates of pseudo-labels.

In Tables 1 and  3, we display experimental results of the segmentation performance of the proposed method on two datasets. As observed in the table, the addition of unlabeled data in CL and CUPL increases the segmentation accuracy in terms of the Dice coefficient and Jaccard index. It is also evident that even with a few numbers of the labeled dataset, such as 50 in Kvasir-SEG, the model achieves higher segmentation accuracy than the baseline (200 labeled set). Similar results were also found in Table 3. Further, we also witness performance increment when introducing the varying number of unlabeled images while keeping fixed labeled images. As the model was trained with the combination of supervised and unsupervised losses, our method takes advantage by leveraging the unlabeled data and propagating the unlabeled data information to the labeled data using a consistency training approach. With this, the model forces a consistent prediction under different augmentation strategies, which eventually helps in better generalization even when the amount of labeled datasets is low. Hence, it proves that the proposed method also works better when the labeled dataset is less.

**Figure 6:**
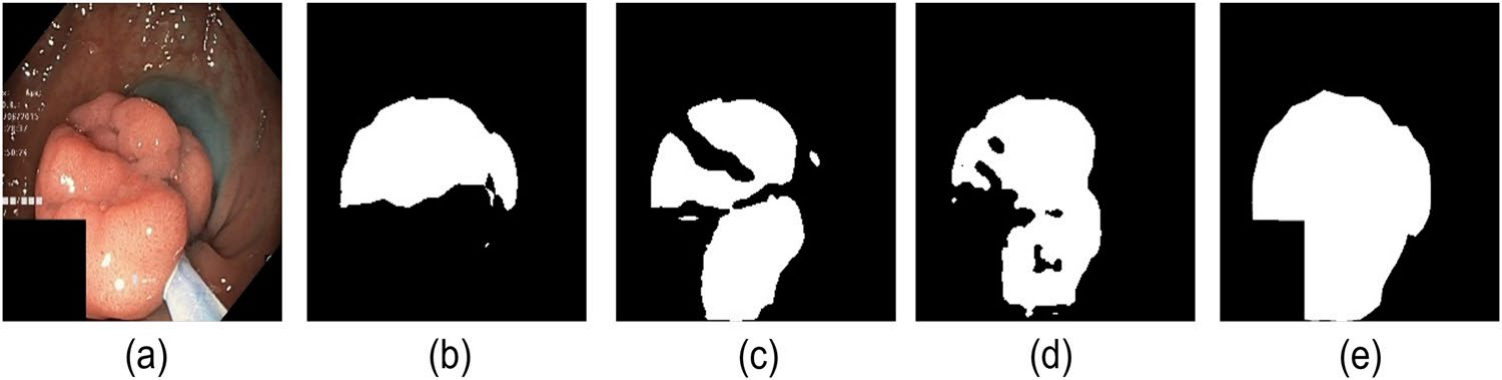
Some pseudo-labels of polyp segmentation obtained by baseline supervised model, Baseline + CL and the baseline + CL + CUPL (Proposed) on the testing subset. Note that the labeled/unlabeled images used for the training was 50/750. Further, (**a–e**) denotes original image, output of “Baseline”, “Baseline + CL”, “Baseline + CL + CUPL (Proposed) ”, and ground truth respectively.

**Figure 7:**
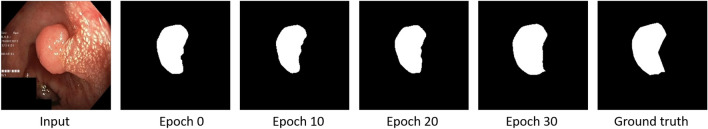
Some pseudo-labels of polyp segmentation obtained by CUPL method in different epochs.

To further test the method’s efficiency, we visualize the output of CL and CUPL, as shown in Fig. 6. It is expected that the segmentation performance is affected by the few labeled datasets. However, we improved the segmentation results on both datasets after adding the unlabeled set into the training dataset by applying the consistency regularization method. These results suggest that both modules improve segmentation accuracy, and the proposed method can generate satisfactory outputs. We also visualize the pseudo-labels in different epochs during optimization for comparison (see Fig. 7). With the semi-supervised settings, the models are constantly updating the outputs to generate better pseudo-labels.

The limitation of the proposed method is the assumption of the same data distribution for the labeled and unlabeled sets. However, obtaining similar distribution might not be possible in real-time clinical applications. When the unlabeled set comes from the different distributions, there is a high possibility of generating false positives, degrading the overall performance. It is necessary to investigate more trustworthy solutions to mitigate or solve the problems in future work. Similarly, strong data augmentation techniques can be applied in consistency training. However, it may not guarantee success in increasing performance; hence requires more research. Despite having a good frame per second (FPS) of 42 by the proposed method, leveraging a large amount of unlabeled data by applying pseudo-label techniques increases the training time and cost, which may not be efficient sometimes.

## Conclusion

This paper proposes a segmentation method for accurate polyp segmentation in a semi-supervised manner. To increase the polyp segmentation generalization, which has a varying shape, size, and scale, we propose a powerful encoder–decoder-based architecture that obtains better segmentation accuracy than prior architectures. Further, to leverage the unlabeled data and propagate its meaningful hidden information to the model, we utilize the consistency regularization approach and train the network on teacher-student strategy by adding supervised and unsupervised loss. We also upgrade a traditional pseudo-labeling scheme by a continuous update of pseudo-labels to generate better outputs. Extensive experiments demonstrate that the proposed method can remarkably increase the segmentation accuracy in the absence of fewer labeled data. It shows the practical importance in clinical settings and can be applied to other domains.

## Data Availability

The datasets generated and/or analysed during the current study are available in https://datasets.simula.no/kvasir-seg/.

## References

[1] Arnold M (2017). Global patterns and trends in colorectal cancer incidence and mortality. Gut.

[2] Pacal I, Karaboga D, Basturk A, Akay B, Nalbantoglu U (2020). A comprehensive review of deep learning in colon cancer. Comput. Biol. Med..

[3] Poudel S, Kim YJ, Vo DM, Lee S-W (2020). Colorectal disease classification using efficiently scaled dilation in convolutional neural network. IEEE Access.

[4] Poudel S, Lee S-W (2021). Deep multi-scale attentional features for medical image segmentation. Appl. Soft Comput..

[5] Pacal I (2022). An efficient real-time colonic polyp detection with yolo algorithms trained by using negative samples and large datasets. Comput. Biol. Med..

[6] Long, J., Shelhamer, E. & Darrell, T. Fully convolutional networks for semantic segmentation. In *Proceedings of the IEEE Conference on Computer Vision and Pattern Recognition*. 3431–3440 (2015). 10.1109/TPAMI.2016.2572683 27244717

[7] Qadir HA, Solhusvik J, Bergsland J, Aabakken L, Balasingham I (2019). A framework with a fully convolutional neural network for semi-automatic colon polyp annotation. IEEE Access.

[8] Ronneberger, O., Fischer, P. & Brox, T. U-net: Convolutional networks for biomedical image segmentation. in *International Conference on Medical Image Computing and Computer-Assisted Intervention*. 234–241 (Springer, 2015).

[9] Zhou, Z., Siddiquee, M. M. R., Tajbakhsh, N. & Liang, J. Unet++: A nested u-net architecture for medical image segmentation. in *Deep Learning in Medical Image Analysis and Multimodal Learning for Clinical Decision Support*. 3–11 (Springer, 2018). 10.1007/978-3-030-00889-5_1 PMC7329239 32613207

[10] Jha, D. *et al.* ResUNet++: An advanced architecture for medical image segmentation. in *Proceedings of the International Symposium on Multimedia*. 225–230 (2019).

[11] Safarov S, Whangbo TK (2021). A-denseunet: Adaptive densely connected unet for polyp segmentation in colonoscopy images with atrous convolution. Sensors.

[12] Borgli H (2020). Hyperkvasir, a comprehensive multi-class image and video dataset for gastrointestinal endoscopy. Sci. Data.

[13] Lee D-H (2013). Pseudo-label: The simple and efficient semi-supervised learning method for deep neural networks. Workshop on challenges in representation learning. ICML.

[14] Berthelot, D. *et al.* Mixmatch: A holistic approach to semi-supervised learning. *Adv. Neural Inf. Process. Syst.* **32** (2019).

[15] Berthelot, D. *et al.* Remixmatch: Semi-supervised learning with distribution alignment and augmentation anchoring. arXiv preprint arXiv:1911.09785 (2019).

[16] Rasmus, A., Valpola, H., Honkala, M., Berglund, M. & Raiko, T. Semi-supervised learning with ladder networks. arXiv preprint arXiv:1507.02672 (2015).

[17] Laine, S. & Aila, T. Temporal ensembling for semi-supervised learning. arXiv preprint arXiv:1610.02242 (2016).

[18] Tarvainen, A. & Valpola, H. Mean teachers are better role models: Weight-averaged consistency targets improve semi-supervised deep learning results. arXiv preprint arXiv:1703.01780 (2017).

[19] Grandvalet, Y., Bengio, Y. *et al.* Semi-supervised learning by entropy minimization. in *CAP*. 281–296 (2005).

[20] Qiao, S., Shen, W., Zhang, Z., Wang, B. & Yuille, A. Deep co-training for semi-supervised image recognition. in *Proceedings of the European Conference on Computer Vision (ECCV)*. 135–152 (2018).

[21] Li, W. *et al.* Semi-supervised learning based on generative adversarial network: A comparison between good gan and bad gan approach. in *CVPR Workshops* (2019).

[22] Souly, N., Spampinato, C. & Shah, M. Semi supervised semantic segmentation using generative adversarial network. in *Proceedings of the IEEE International Conference on Computer Vision*. 5688–5696 (2017).

[23] Baheti, B., Innani, S., Gajre, S. & Talbar, S. Eff-unet: A novel architecture for semantic segmentation in unstructured environment. in *Proceedings of the IEEE/CVF Conference on Computer Vision and Pattern Recognition Workshops*. 358–359 (2020).

[24] Gu Z (2019). Ce-net: Context encoder network for 2D medical image segmentation. IEEE Trans. Med. Imaging.

[25] Li, Z., Pan, J., Wu, H., Wen, Z. & Qin, J. Memory-efficient automatic kidney and tumor segmentation based on non-local context guided 3D u-net. in *International Conference on Medical Image Computing and Computer-Assisted Intervention*. 197–206 (Springer, 2020).

[26] Wang, W., Zhong, J., Wu, H., Wen, Z. & Qin, J. Rvseg-net: An efficient feature pyramid cascade network for retinal vessel segmentation. in *International Conference on Medical Image Computing and Computer-Assisted Intervention*. 796–805 (Springer, 2020).

[27] Zhong, J., Wang, W., Wu, H., Wen, Z. & Qin, J. Polypseg: An efficient context-aware network for polyp segmentation from colonoscopy videos. in *International Conference on Medical Image Computing and Computer-Assisted Intervention*. 285–294 (Springer, 2020).

[28] Fan, D.-P. *et al.* Pranet: Parallel reverse attention network for polyp segmentation. arXiv preprint arXiv:2006.11392 (2020).

[29] Huang, C.-H., Wu, H.-Y. & Lin, Y.-L. Hardnet-mseg: A simple encoder-decoder polyp segmentation neural network that achieves over 0.9 mean dice and 86 fps. arXiv preprint arXiv:2101.07172 (2021).

[30] Chao, P., Kao, C.-Y., Ruan, Y.-S., Huang, C.-H. & Lin, Y.-L. Hardnet: A low memory traffic network. in *Proceedings of the IEEE/CVF International Conference on Computer Vision*. 3552–3561 (2019).

[31] Masood, A., Al-Jumaily, A. & Anam, K. Self-supervised learning model for skin cancer diagnosis. in *2015 7th International IEEE/EMBS Conference on Neural Engineering (NER)*. 1012–1015 (IEEE, 2015).

[32] Gu, L. *et al.* Semi-supervised learning for biomedical image segmentation via forest oriented super pixels (voxels). in *International Conference on Medical Image Computing and Computer-Assisted Intervention*. 702–710 (Springer, 2017).

[33] Jaisakthi, S., Chandrabose, A. & Mirunalini, P. Automatic skin lesion segmentation using semi-supervised learning technique. arXiv preprint arXiv:1703.04301 (2017).

[34] Bai, W. *et al.* Semi-supervised learning for network-based cardiac mr image segmentation. In *International Conference on Medical Image Computing and Computer-Assisted Intervention*, 253–260 (Springer, 2017).

[35] Hung, W. C., Tsai, Y. H., Liou, Y. T., Lin, Y. Y. & Yang, M. H. Adversarial learning for semi-supervised semantic segmentation. in *29th British Machine Vision Conference, BMVC 2018* (2019).

[36] Zhang, Y. *et al.* Deep adversarial networks for biomedical image segmentation utilizing unannotated images. in *International Conference on Medical Image Computing and Computer-Assisted Intervention*. 408–416 (Springer, 2017).

[37] Nie, D., Gao, Y., Wang, L. & Shen, D. Asdnet: attention based semi-supervised deep networks for medical image segmentation. in *International Conference on Medical Image Computing and Computer-Assisted Intervention*. 370–378 (Springer, 2018).

[38] Hou, J., Ding, X. & Deng, J. D. Semi-supervised semantic segmentation of vessel images using leaking perturbations. in *Proceedings of the IEEE/CVF Winter Conference on Applications of Computer Vision*. 2625–2634 (2022).

[39] Athiwaratkun, B., Finzi, M., Izmailov, P. & Wilson, A. G. There are many consistent explanations of unlabeled data: Why you should average. arXiv preprint arXiv:1806.05594 (2018).

[40] Cui, W. *et al.* Semi-supervised brain lesion segmentation with an adapted mean teacher model. in *International Conference on Information Processing in Medical Imaging*. 554–565 (Springer, 2019).

[41] Luo, X., Chen, J., Song, T. & Wang, G. Semi-supervised medical image segmentation through dual-task consistency. arXiv preprint arXiv:2009.04448 (2020).

[42] Zhang, Y., Zhou, B., Chen, L., Wu, Y. & Zhou, H. Multi-transformation consistency regularization for semi-supervised medical image segmentation. in *2021 4th International Conference on Artificial Intelligence and Big Data (ICAIBD)*. 485–489 (IEEE, 2021).

[43] Zhou H-Y (2021). Ssmd: Semi-supervised medical image detection with adaptive consistency and heterogeneous perturbation. Med. Image Anal..

[44] Jha, D. *et al.* Kvasir-seg: A segmented polyp dataset. in *International Conference on Multimedia Modeling*. 451–462 (Springer, 2020).

[45] Li, X. *et al.* Transformation-consistent self-ensembling model for semisupervised medical image segmentation. *IEEE Transactions on Neural Networks and Learning Systems* (2020). 10.1109/TNNLS.2020.2995319 32479407

[46] Sajjadi, M., Javanmardi, M. & Tasdizen, T. Regularization with stochastic transformations and perturbations for deep semi-supervised learning. arXiv preprint arXiv:1606.04586 (2016).

[47] Tan, M. & Le, Q. V. Efficientnet: Rethinking model scaling for convolutional neural networks. arXiv preprint arXiv:1905.11946 (2019).

[48] Sandler, M., Howard, A., Zhu, M., Zhmoginov, A. & Chen, L.-C. Mobilenetv2: Inverted residuals and linear bottlenecks. in *Proceedings of the IEEE Conference on Computer Vision and Pattern Recognition*. 4510–4520 (2018).

[49] Hu, J., Shen, L. & Sun, G. Squeeze-and-excitation networks. in *Proceedings of the IEEE Conference on Computer Vision and Pattern Recognition*. 7132–7141 (2018).

[50] Chen, L. *et al.* Sca-cnn: Spatial and channel-wise attention in convolutional networks for image captioning. in *Proceedings of the IEEE Conference on Computer Vision and Pattern Recognition*. 5659–5667 (2017).

[51] Bernal, J. *et al.* Wm-dova maps for accurate polyp highlighting in colonoscopy: Validation vs. saliency maps from physicians. *Comput. Med. Imaging Graph.* **43**, 99–111 (2015). 10.1016/j.compmedimag.2015.02.007 25863519

